# The Effects of Therapeutic Ultrasound on Human Milk Composition: A Quasi-Experimental Pre-Post Design Study

**DOI:** 10.1177/08903344261430154

**Published:** 2026-03-29

**Authors:** Leanda Jane McKenna, Kristin Eu, Renee McGregor, Stephanie Melanko, Aik Ping Tay, Patricia Gaunt, Ching Tat Lai, Donna Tracy Geddes, Angela Jacques, Adelle McArdle

**Affiliations:** 1 Curtin University, Perth, WA, Australia; 2 Physiotherapy Department, King Edward Memorial Hospital, Perth, WA, Australia; 3 The University of Western Australia, Crawley, WA, Australia; 4 Notre Dame University, Fremantle, WA, USA; 5 Monash University, Churchill, VIC, Australia

**Keywords:** breastfeeding, exclusive breastfeeding, human milk - biochemistry, lactation management, mastitis, pre-post design, therapeutic ultrasound, treatment

## Abstract

**Background:**

Therapeutic ultrasound generates heating within tissues and is a popular treatment option for inflammatory conditions of the lactating breast, but it is unknown if therapeutic ultrasound alters breastmilk composition.

**Research Aim:**

This study aimed to determine the effect of therapeutic ultrasound on the protein, lactose and fat concentrations of human milk in healthy lactating mothers.

**Method:**

This was a single session quasi-experimental, non-superiority, pre-post design study. Twenty-six Western Australian mothers with infants aged 1–6 months and exclusively breastfeeding were included. Mothers with inflammatory conditions of the lactating breast or contraindicated from receiving therapeutic ultrasound, were excluded. Therapeutic ultrasound was applied for 10 minutes to the right breast and samples of 5 ml expressed human milk were collected pre and post ultrasound application. Protein, lactose, and fat concentrations were measured by the Bradford method, enzymatic spectroscopy, and creamatocrit. Statistical analysis included general linear mixed modelling.

**Results:**

There was no significant difference in human milk protein composition between measures taken pre and post ultrasound (mean difference, -0.64g/L; 95% CI [-1.93, 0.64], *p* = 0.328). There was no significant difference in human milk lactose composition (mean difference, -4.77g/L; 95% CI [-11.57, 2.03], *p* = 0.169) between measures taken pre and post ultrasound. There was a significant increase in human milk fat composition between measures taken pre and post ultrasound (mean difference, 1.36%; 95% CI [0.97, 1.75], *p* <0.001).

**Conclusion:**

Therapeutic ultrasound applied to the healthy lactating breast does not adversely affect the protein, lactose, or fat concentrations of human milk.

## Introduction

Inflammatory conditions of the lactating breast (ICLB) cover a spectrum of breast pathologies including engorgement, blocked ducts, mastitis, and breast abscess, thought to be associated with pain, erythema, and flu-like symptoms (Aktimur et al., 2016; Gianni et al., 2019; Heron et al., 2020; Mitchell et al., 2022). These symptoms can affect up to 33% of breast-feeding mothers in the early postpartum period and can contribute to early cessation of breastfeeding (Aktimur et al., 2016; Gianni et al., 2019). Health care interventions for the treatment of ICLB may include hot and/or cold compresses, patient education, specific manual techniques and therapeutic ultrasound (TUS; Cooper & Kowalsky, 2015; Lavigne & Gleberzon, 2012). Therapeutic ultrasound is a recommended intervention for ICLB in the Academy of Breastfeeding Medicine mastitis clinical protocol (Mitchell et al., 2022). Therapeutic ultrasound is used in 91% of occasions to Australian women who received physiotherapy care for ICLB management (Diepeveen et al., 2019).

Therapeutic ultrasound can produce thermal and non-thermal effects, through sound wave energy. This may assist in resolving ICLB by reducing inflammation, accelerating tissue healing, clearing blocked ducts and other antimicrobial properties (Cooper & Kowalsky, 2015). Therapeutic ultrasound has been found to create heating in muscle in therapeutic applications (Weaver et al., 2006), and some mothers find that breast heating can be significant with TUS (Neill et al., 2025). Given that heating used to remove potentially harmful bacteria can reduce protein concentration in human milk banks (Peila et al., 2016), it is currently unknown if the heating associated with TUS may alter breast milk composition.

Human milk is the optimal nutritional source for infants, and its benefits include protection from infectious agents, higher levels of intelligence, and lower rates of diarrhea, respiratory illness, and obesity. Breastfeeding offers maternal protective effects from breast cancer, some gynaecologic cancers and cardiovascular disease (Victora et al., 2016). Human milk contains numerous important components, the most abundant of these being water (87%), lactose (7%), total fat (4%) and total protein (1%; Butts et al., 2018). Protein composition includes both nutritive and non-nutritive proteins that support child growth and development, protect against pathogens, and contribute to immune function. Lactose is a carbohydrate that accounts for 85% of total carbohydrates for infants and is an important energy source. It is an osmotic driver of milk production and possesses antibacterial effects. Fat contributes to at least 50% of the infant’s caloric intake and contains lipid species that support neurodevelopment and cognition (George et al., 2021; Peila et al., 2016; Zhu & Dingess, 2019).

Given these important functions of human milk, research must identify whether human milk composition is adversely affected by TUS to ensure that ICLBs are effectively treated without compromising infant outcomes. This allows health care professionals to deliver patient-centred care by providing evidence-based information on the benefits and risks of TUS to breastfeeding mothers who may require TUS for the treatment of ICLB.

**Key Messages**

- Therapeutic ultrasound generates heating within tissues and is a popular treatment option for inflammatory conditions of the lactating breast, but it is unknown if therapeutic ultrasound alters breastmilk composition.
- Therapeutic ultrasound applied to the healthy lactating breast does not adversely affect the protein, lactose, or fat composition of breastmilk.
- Healthcare professionals who recommend therapeutic ultrasound to treat mothers who have inflammatory conditions of the lactating breast can provide greater reassurance to mothers that therapeutic ultrasound does not affect the nutrient value of human milk and therefore should not affect breastfeeding babies.

The primary aim of this study was to determine the effect of TUS administered to the healthy lactating breast on the total protein, fat, and lactose concentration within human milk. The primary hypothesis of this study was that total protein, lactose, and total fat concentrations within human milk would not change significantly after the application of TUS to the breast.

## Methods

### Research Design

The study was a quasi-experimental pre-post design conducted in a single session. This research design was chosen to minimize impact on the mother, and maximize recruitment by using a single session. This study was registered with the Australian New Zealand Clinical Trials Registry (ACTRN12621001025820p) and was approved by Curtin University Human Research Ethics Committee (HRE2021-0469). Participants provided written informed consent prior to commencing the study.

### Setting and Relevant Context

This study was conducted in Australia where 73.5%, 63.9%, and 37.5% of mothers exclusively breastfeed at 2, 4, and 6 months, respectively (Australian Bureau of Statistics, 2023). Australian mothers are encouraged to exclusively breastfeed to 6 months (Australian Government Department of Health Disability and Ageing, 2019), with government strategies focused on increasing the number of breastfeeding friendly environments in workplaces, public spaces, health services, and early childhood education centers (Australian Government Department of Health Disability and Ageing, 2019). Free healthcare is available to all the population and approximately half (Australian Prudential Regulation Authority, 2024) of the population has private health insurance, which can be used to access physiotherapy for treatment of an ICLB. At the time of the study Australian mothers were entitled to 20 weeks of paid parental leave.

### Sample

The target population were Western Australian mothers who were exclusively breastfeeding (as defined by the World Health Organization (WHO, 2023)) and were recruited via word-of-mouth and social media promotional posts that contained an online Qualtrics (Qualtrics, Utah, USA) survey to determine eligibility. Word-of-mouth recruitment and social media posts utilized researchers and university connections. Mothers were eligible to participate if they were over 18 years old, with infants aged 1–6 months at data collection. Mothers who were mixed feeding with formula and solids were excluded. Mothers with a history and/or current ICLB for their currently breastfed baby who received laser or ultrasound treatment were excluded. Mothers were excluded if they had a current pregnancy, known or suspected cancer, circulation problems, bleeding disorders, acute infection, disease of the nervous system, heart conditions, possessed cardiac pacemakers, or any other electronic/metallic/breast implants as these contraindicate the use of TUS. Mothers did not receive any compensation.

Power calculations were conducted using G*power freeware (Faul et al., 2009), based on the primary outcome measure of human milk protein concentration. Additionally, in comparison to lactose or fat, protein is more likely to be impacted by TUS, due to potential denaturing of proteins (Peila et al., 2016). For a within factors repeated measures ANOVA, *n* = 22 was required to detect an effect size *f* = 0.35 (equivalent to a partial eta-squared of 0.11, based on a 10% change in protein from a mean of 12.6g/L with pooled *SD* = 1.8) within a single group over 2 timepoints (pre-post), with alpha = 0.025 as per a non-inferiority study. Based on a 10% contingency, a sample of *n* = 25 was estimated. A sample size of 26 mothers was obtained and being larger than the a priori required estimate, is adequate.

### Measurements

Mothers were asked to complete a demographics survey via Qualtrics, which determined the mother’s age (years), current number of children, baby’s age (months) and time since the right breast was drained. The baby’s age was used to approximate lactation stage. Over-bust and under-bust bra measurements were then taken using a soft, cloth tape measure with the bra on (Spencer et al., 2019).

Continuous TUS (Chattanooga, model: Intelect Advanced 2772MC, Texas, USA) was applied to the right breast using a 5 cm^2^ soundhead for 10 minutes at 1MHz and 1.8 Wcm^2^. This intensity is the most used intensity for ICLB treatment within Australia (Diepeveen et al., 2019). One researcher (RM), a qualified physiotherapist with experience in application of TUS, applied the TUS to the right breast of each participant once in a single session. The soundhead was applied closely adjacent to the nipple, over the inferolateral region of the right breast. This area was chosen as it has greater amounts of glandular tissue and is more susceptible to ICLB (Cox et al., 1996; Geddes, 2007). The soundhead movement was applied from a superolateral to inferomedial direction to ensure that the collected milk was most likely to be exposed to the TUS. This targeted the branches of the milk ducts from the glandular tissue as they converge into a main collecting duct near the nipple (Geddes, 2007; Rusby et al., 2007).

The protein content in the milk samples was determined by a modified Bradford method using a commercial protein reagent (Bio-Rad Laboratories, USA; Bradford, 1976). Protein standards were prepared from an aliquot of human milk and the protein concentration determined by the Kjeldhal method, as described previously (Atwood & Hartmann, 1992), and the protein assay, as described previously (Mitoulas et al., 2002). The recovery of a known amount of protein added to the milk samples was 98 ± 6% (*n* = 6). The detection limit of this assay was 0.035g/L and the inter-assay CV was 3% (*n* = 2). The Bradford protein assay, a spectrophotometric method, has the highest sensitivity (correlation of 0.97 with gold standard; Lonnerdal et al., 1987) for protein, making it valid for use in our study.

The concentration of lactose in the milk sample was determined by an enzymatic spectroscopic method (Arthur et al., 1989; Kleyn, 1985). The recovery of a known amount of lactose added to the milk samples was 99 ± 2.8% (*n* = 7). The detection limit of this assay was 2.02g/L and the inter-assay CV was 10% (*n* = 2). This enzymatic method was used due to its sensitivity, specificity (mean difference to other methods of 0.04%), lower cost, speed, and ease of analysis (Kleyn, 1985).

Fat composition in the milk samples were determined by the creamatocrit method (Du et al., 2017; Lucas et al., 1978). Frozen milk samples were thawed at 37^o^C (98.6 ^o^F) for 1 hr prior to the fat measurement. The thawed samples were analyzed with Intelli-mixer (RM-2, ELMI Ltd, Riga, Latvia) using UU mode for 15 s at 50 rpm and followed by three gentle top-to-bottom inversions. The mixed milk was sampled in duplicate using glass capillary-tubes (41A2502, Kimble-Chase, New Jersey, USA), sealed one end with tube sealing compound (43510, Kimble-Chase, New Jersey, USA), and spun in a flat-bed centrifuge (CEN 96221, Phoenix Scientific Industries Ltd, Phoenix, USA) designed for capillary-tubes for 10 min. Spun capillary-tubes were read on Creamatocrit Plus™ (Medela, AG, Baar, Switzerland) machine to determine the fat content in percentage using the formula: fat content = 3.968 + (5.917 x creamatocrit [%]; Lucas et al., 1978) The skim milk portion in the glass capillary was obtained by cutting off the capillary with a glass cutter and was transferred to a new clean tube. It was then used for protein and lactose assays. The creamatocrit analysis has shown strong correlation (r^2^ = 0.99) with the gold standard labor intensive gravimetric method to analyze fat content in milk (Du et al., 2017).

The data from mothers that could not tolerate TUS at 1.8 cm^2^ was removed from the data set after analysis by the lab technician. The lab technician was unaware of which data met research protocol and which did not and thus could not bias results. Additionally, the results were primarily derived by machine assessment with minimal human bias.

### Data Collection

Data collection occurred in an enclosed room within a private facility in Perth, Western Australia. from August to October 2021. Participants who met inclusion criteria from the eligibility survey were contacted to arrange an appointment for them to participate. Mothers were informed of the possibility of experiencing heating effects from the TUS before gaining written informed consent. Mothers were asked not to breastfeed for at least 2 hours before the application of TUS.

Pre-TUS milk samples were collected immediately before the intervention. Milk samples were collected via hand expression or an electric breast pump (Medela, model: Symphony, Victoria, Australia) from the right breast into a 5 ml polypropylene plastic vial. Most mothers were able to hand express into the plastic vial, without assistance from the researchers. The milk samples were labelled as either “pre-TUS" or “post-TUS” with the mother’s allocated identification number, time, and date of data collection. Mothers were positioned in a left side-lying position, so that the right breast would rest in a symmetrical, conical shape when TUS was applied. Post-TUS milk samples were collected immediately after. These were then stored in a -20^o^C (-4 ^o^F) freezer located within the building. Once all the milk samples were collected, they were transported on ice to the laboratory at ** where they were stored in a -20^o^C (-4 ^o^F) freezer prior to biochemical analysis.

### Data Analysis

Results were summarized using marginal means and 95% confidence intervals. Generalized linear mixed models were used to analyze pre-post differences in human milk composition, after adjusting for lactation stage as determined by age of the baby and time since last feed from the right breast in minutes. Individual level data was included in the models and presented as group summaries. Statistical analysis was completed using Stata (Version 16.0).

## Results

**Figure 1.**
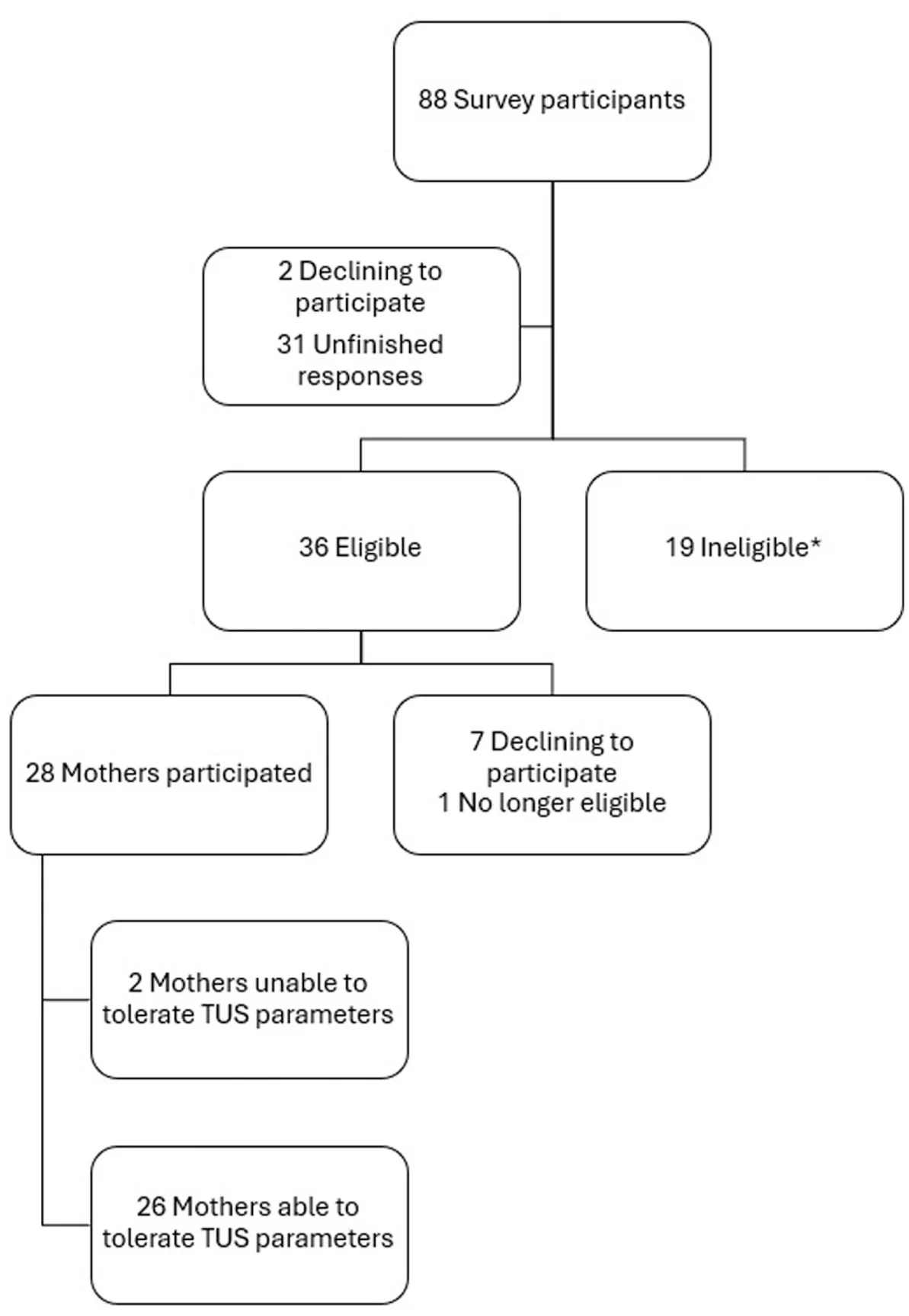
Diagram describing the flow of participants into the study. *Ineligible due to mixed feeding or received ultrasound/laser for the current breastfed child or had contraindications to TUS.

Data from 26 mothers who were exclusively breastfeeding and able to tolerate TUS parameters at 1.8 Wcm^2^ were analyzed; two mothers were excluded as they could only tolerate TUS parameters at 1.5 Wcm^2^ (Figure 1). Participant demographics are displayed in Table 1.

**Table 1.** Participant Characteristics (*N* = 26).

	Median	Q1, Q3	Min–Max
Number of children	2	1, 2	1–3
	Mean	*SD*	Min–Max
Age (years)	31.7	3.6	26–40
Over bust (cm)	102.2	12.6	80.5–132.0
Under bust (cm)	88.1	11.8	69.0–120.0
Lactation stage (months)^a^	2.3	1.3	1–5
Time since last feed (min)^a^	159.0	58.9	120–360

*Note.* Q = quartile.

a Lactation stage and time since the last feed were controlled for during the generalized linear mixed models.

**Figure 2.**
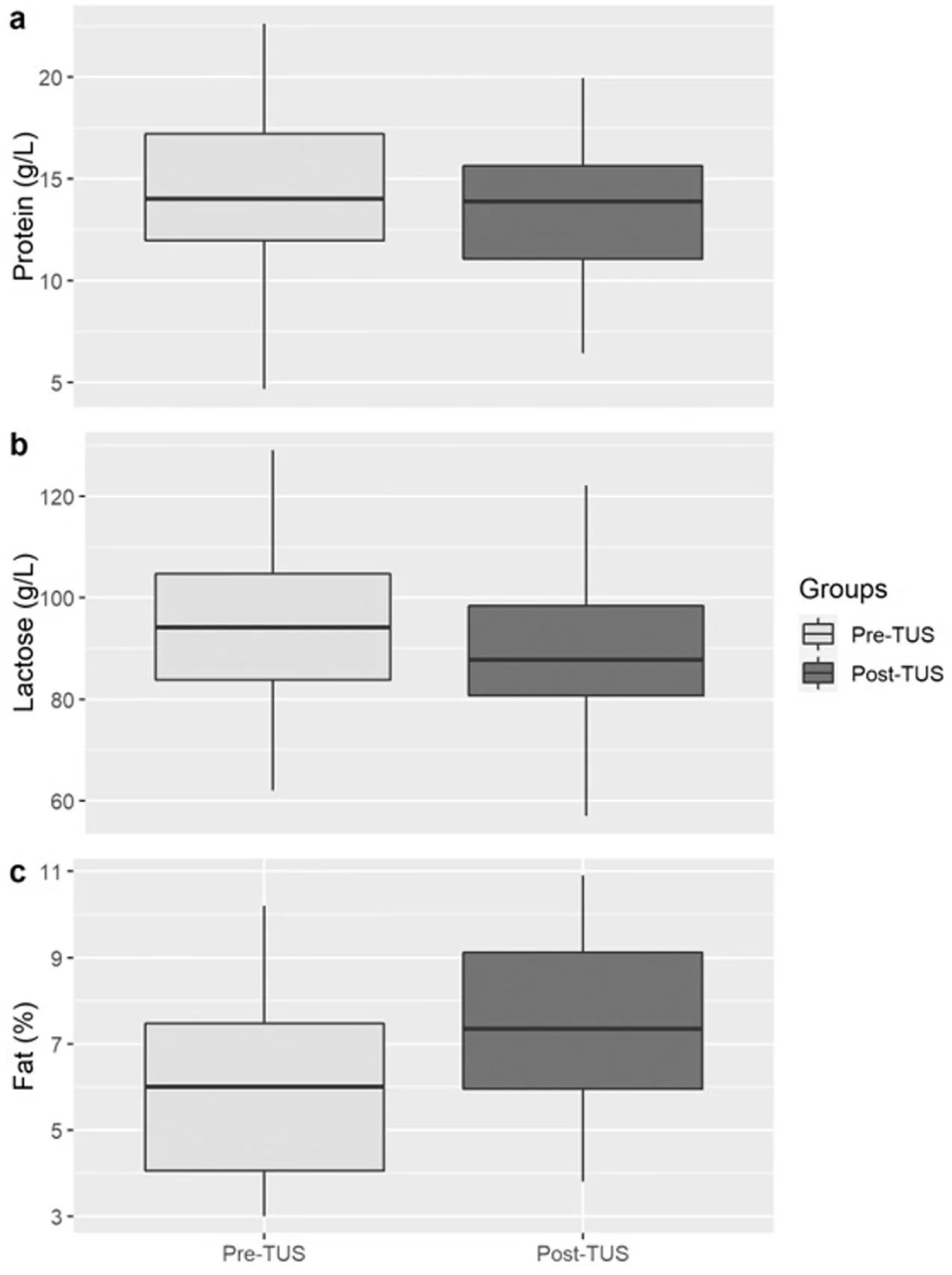
Box Plot Graphs Showing Concentrations of (a) Protein, (b) Lactose and (c) Fat Pre and Post Therapeutic Ultrasound. *Note.* Pre-TUS = pre application of therapeutic ultrasound; Post-TUS = post application of therapeutic ultrasound; g/L= grams per litre.

The results for human milk composition before and after application of TUS are displayed in Table 2 and Figure 2 There was no significant change in protein composition between pre-TUS and post-TUS milk samples (mean difference, -0.64g/L; 95% CI [1.93, 0.64], *p* = 0.328) or lactose composition (mean difference, -4.77g/L; 95% CI [11.57, 2.03], *p* = 0.169). A significant increase in fat composition was found between the pre and post samples (mean difference, 1.36%; 95% CI [0.97, 1.75], *p* < 0.001).

**Table 2.** Adjusted Protein, Lactose and Fat Composition Measures Pre and Post Therapeutic Ultrasound.

	Pre		Post		Change
	Mean	95% CI	Mean	95% CI	*p*
Protein (g/L)	14.3	12.9, 15.7	13.6	12.2, 15.0	0.328
Lactose (g/L)	93.7	87.9, 99.5	88.9	83.1, 94.7	0.169
Fat (%)	6.0	5.2, 6.7	7.3	6.6, 8.1	< 0.001

## Discussion

Therapeutic ultrasound administered to the healthy lactating breast did not result in a significant change in the concentration of protein within human milk. Lactose concentration also remained unchanged after application of TUS. There was a small increase in fat concentration. These results indicate that the application of TUS does not adversely affect the examined macronutrient concentration of human milk. This is believed to be the only study to examine the thermal effects of 10 minutes of TUS treatment, on in vivo human milk composition.

This result contrasts with previous research that has examined the effect of heating on human milk protein in vitro, via the Holder pasteurization method—a method that heats milk to 62.5°C (144.5^o^F) for 30 minutes and then cools it to room temperature to remove potentially harmful bacteria, for use in human milk banks (Peila et al., 2016). The protein concentration in the prior study was reduced, which is plausible given that the human milk was heated to 62.5°C (144.5^o^F) for 30 minutes (Peila et al., 2016), rather than the in vivo 10-minute TUS duration seen in this study. It is not believed that TUS, either in our current study or in clinical practice, could create this temperature increase within the breast tissue seen in the Holder pasteurization method. While such a conclusion is yet to be conclusively confirmed with research, studies of TUS application to muscles have shown a heating effect of 43°C ± 2.0°C (109.4°F ± 35.6°F) at an intensity of 1.5 W/cm2 (Weaver et al., 2006). Regarding lactose concentration, the prior study that utilized the Holder pasteurization method did not find a change in lactose (Peila et al., 2016). The results from the present study are consistent with these prior findings.

We detected a small but significant increase in the fat concentration between pre- and post-TUS samples. During normal physiological human milk removal, fat globules from the alveoli are displaced, causing an increase in total fat concentration (Hassiotou et al., 2013). A wide variation of change in total fat has been reported, and is thought to occur with removal of as little as 5 ml of human milk (Hassiotou et al., 2013). This may account for the increase in fat concentration in human milk post TUS application in the present study, as 10 ml of milk in total was removed from the breast. Therefore, it cannot be concluded that the application of TUS is the cause of the increase in fat concentration. It can, however, be established that the macronutrients are not adversely affected. Human milk fat is important for the development of infant central nervous system, immunity, and inflammatory responses (Perrella et al., 2021), thus an increase in fat concentration would not be considered an adverse effect.

The biggest strength of this study was its clinical translatability. The parameters used in the application of TUS align with those most frequently used by physiotherapists (Diepeveen et al., 2019). The location and size of the treatment area was standardized, and the same researcher applied the TUS to all subjects. Further strengths of this study include the analysis of all milk samples in the same batch, reducing the possibility of inter-assay variability. The pre-post design of this study was chosen to decrease the impact of potential confounding variables, as the participants acted as their own controls. Pre and post samples were collected immediately either side of the intervention, also reducing confounding.

## Limitations

Some limitations in this study arise from the study design. The lack of a placebo-based control group does not allow for the comparison with normal changes in macronutrient concentration as the breast empties. However, prior research provides good indication of these normal changes, and the pre-post design allows for within-subject control. The present study applied TUS to the inferolateral region of the right breast but analyzed milk from the entire right breast, potentially diluting the effect of the TUS. It is recognized that direct temperature measurements within the breast were not undertaken, and thus the temperature that the human milk was subjected to is unknown. However, in-breast temperature measurement is ethically complex and technically challenging, and noninvasive methods such as thermography, skin sensors, or thermal modeling are unlikely to reflect deeper temperatures. Studies have demonstrated that the correlation between thermography and gold standard thermocouple measurement at the skin surface is weak (De Andrade Fernandes et al., 2014); thus, conclusions about deeper tissue temperatures cannot be dependably drawn from skin temperature measurements. The application of TUS over surface temperature sensors would impede the transmission of TUS into the breast tissue, confounding the treatment effect. In addition, thermal modeling would be affected by the individual’s metabolic heat generation, skin fat thickness, blood flow, differences in thermal conductivities, size and shape of glandular tissue, fat tissue, human milk, and lactiferous ducts (Agrawal & Pardasani, 2016). However, irrespective of the temperature within the breast, this study provides information of the effect of TUS on protein, lactose, and fat. While protein, lactose, and fat are major nutritional components, human milk contains numerous bioactive molecules that play critical roles in infant immune development (Perrella et al., 2021). Other human milk components such as secretory IgA (Mayayo et al., 2016), lactoferrin, lysozyme, and cytokines may be more sensitive to denaturation than macronutrients (Buffin et al., 2018; Daniels et al., 2017), and further studies should investigate these. Generalizability of the results could be affected, as our study included only healthy lactating mothers. Given that ICLB itself alters human milk composition, this study provides information that TUS does not appear to affect healthy human milk, aiding the understanding of how TUS interacts with human milk. TUS is used as a physiotherapy technique in the treatment of ICLB where there is inflamed breast tissue, thus, our study is partially representative of the clinical population that would undergo this treatment technique. It would be beneficial to conduct further research into the effects of TUS on human milk composition within the ICLB population specifically, providing evidence that aligns with the clinical population. There remain challenges with such a study as careful consideration is required, given how unwell mothers with ICLB can be, the resulting time burden on unwell mothers with infants, and the proven difficulty in recruiting mothers to research who have a breast pathology (Amir et al., 2004).

## Conclusions

Given the importance of human milk quality in child development, the results of our study are reassuring. Healthcare professionals who use TUS to treat mothers who have an ICLB can provide greater reassurance to mothers that TUS does not affect the nutrient value of human milk, and therefore should not affect breastfeeding babies. This study also provides reassurance to researchers that TUS does not affect human milk, providing ethical basis for further research into physiotherapy interventions for ICLB.
